# Dismal situation of cardio pulmonary resuscitation knowledge and skills among junior doctors in twin cities of Pakistan

**DOI:** 10.12669/pjms.35.5.785

**Published:** 2019

**Authors:** Sanniya Khan Ghauri, Arslaan Javaeed, Faiza Shah, Misbah ul Hasan Ghani

**Affiliations:** 1 Sanniya Khan Ghauri, MBBS, MRCEM. Department of Emergency Medicine, Shifa International Hospital, Islamabad, Pakistan; 2 Arslaan Javaeed, MBBS, M.Phil, MHPE. Poonch Medical College, Rawalakot, Azad Kashmir, Pakistan; 3 Faiza Shah, MBBS. Poonch Medical College, Rawalakot, Azad Kashmir, Pakistan; 4 Misbah ul Hasan Ghani. MBBS, MPH. Poonch Medical College, Rawalakot, Azad Kashmir, Pakistan

**Keywords:** KAP, CPR, Pakistan, Doctor, BLS

## Abstract

**Objective:**

To assess the knowledge, attitude, and practice of cardio pulmonary resuscitation (CPR) among junior doctors in 13 tertiary care hospitals of Rawalpindi and Islamabad.

**Methods:**

A total of 317 junior doctors from 13 tertiary care hospitals in Rawalpindi and Islamabad in Pakistan were included in this cross-sectional study. Data were collected using a 37-item interviewer-administered structured questionnaire by the researchers. Informed consent and ethical approval were secured. Doctors’ knowledge, attitude, and practice regarding CPR were presented and compared across the demographic variables (age, gender, CPR training etc.). Data analysis was done using SPSS V 23 at an alpha level of 5%.

**Results:**

Response rate for this study was 87.08%. Abbreviations of BLS, AED, and EMS were known by 94.3%, 36.0%, and 41.0% doctors respectively. No doctor had complete knowledge of CPR. Less than half of the participants knew the proper compression depths. Overall knowledge regarding CPR steps was poor. Out of 31 CPR knowledge, attitude, and practice related questions 21 correct answers were given by two doctors which was the highest score. The mean KAP score was 14.18 ± 0.15.

**Conclusion:**

Awareness regarding CPR is essential for all doctors. Many authorities in developed countries are giving CPR training to the general population whereas in Pakistan, many of the doctors never had CPR training. The current study showed the clear majority wants hands-on CPR training. Hospital authorities may find this as an opportunity to improve the knowledge and skills of health workers.

## INTRODUCTION

Cardiovascular diseases (CVDs) are the number one cause of death globally.^1^ According to the World Health Organization, about 17.3 million people died from Cardiovascular Diseases (CVDs) in 2008. This represented 30% of all the global deaths.^2^ Three-quarters of all deaths from myocardial infarction occur after cardiac arrest in the community.^3^ This proportion is even higher in people under 55 years of age, in whom 91% of cardiac arrest deaths occur out of the hospital.^3^ In these conditions, early Cardio Pulmonary Resuscitation (CPR) and early defibrillation might be useful to improve the survival and neurologic outcomes.^4^

Basic life support (BLS) is the foundation for saving lives following cardiac arrest. Fundamental aspects of BLS include immediate recognition of sudden cardiac arrest (SCA) and activation of the emergency response system, early cardiopulmonary resuscitation (CPR), and rapid defibrillation with an automated external defibrillator (AED).^5^ All healthcare professionals are expected to have current knowledge of Basic Life Support (BLS) guidelines to revive unresponsive and cardiac arrest patients.^6^

In the present study, we aimed to assess the knowledge, attitude, and practice about BLS among the doctors of 13 tertiary care hospitals in Rawalpindi and Islamabad, Pakistan.

## METHODS

Junior doctors from 13 tertiary care hospitals of Rawalpindi and Islamabad, Pakistan were included in this study as the respondents. The doctors who have been working for less than four years in a hospital were considered as junior doctors. The sample size was calculated using widely used formulae z^2^*p(1-p)/e^2^.^1^ Since the prevalence rate was unknown, 50% prevalence was considered. At an alpha level of 5%, the required sample size was 384. In order to include the required samples, the researchers visited these hospitals during the morning, evening and night shifts to recruit as many junior doctors as possible. During the study duration, 364 junior doctors were inducted in the study out which 317 returned the questionnaire with a response rate of 87.08%. The data was collected from April 2018 to October 2018 through a 37 items questionnaire related to demographic characteristics, knowledge, attitude, and practice of CPR among junior doctors. The questionnaire validation was done by two epidemiology professors. Researchers interviewed all the included doctors face to face with the questionnaire. Purpose of the current study was clearly explained to the interviewees. Informed consent was taken from each participant. Ethical approval was secured from Institutional Review Board of Poonch Medical College, Rawalakot, Azad Kashmir, Pakistan.

### Statistical Analysis

Frequencies and percentages were used to present the demographic characteristics and CPR knowledge, attitude, and practice related questions and responses. The mean ± SD number of correct responses to all CPR related questions were presented. The mean number of correct answers were compared between genders, doctor having CPR training (yes/ no), and doctors attended CPR course (yes/ no) by Mann Whitney U test. The mean number of correct responses were compared across the age groups, time since graduation and time since last CPR training by Kruskal Wallis H test.

The analysis was performed in 95% confidence interval using the Statistical Package for Social Science (SPSS), version 23.0 (IBM, Armonk, NY, USA).

## RESULTS

Among the 317 total respondents, 171 (53.9%) were male and 258 (81.4%) were from age group 23 to 25 years. More than half had (54.6%) valid CPR training certificate. (Table-I)

**Table 1:** Demographic characteristics of all respondents.

Characteristics	N	%
***Age of the doctors***		
23-25	258	
26-30	52	
31-34	7	2.2
***Gender of doctors***		
Male	146	
Female	171	53.9
***Time since graduation***		
Last year	268	
Last 2-3 years	21	6.6
Last 4-5 years	12	3.8
> 5 years	16	5.0
Doctor having valid CPR certificate	173	54.6
***Time since last CPR training***		
1 year or less	264	83.3
>1 to 2 years	31	9.8
>2 to 3 years	9	2.8
>3 to 4 years	13	4.1
Doctors attended CPR course	226	71.3

The majority, 299 (94.3%) knew the abbreviation of ‘BLS’. Ninety-eight percent respondents thought BLS training is necessary for the doctors. More than half (53.0%) knew the correct compression to ventilation ration which is 30:2. Responses to the 13 CPR knowledge related questions, seven CPR attitude related questions and 11 CPR practice related questions were presented in Table-I, Table-II, and Table-III respectively. Mean number of correct answers for all 31 CPR questions for all respondents was 14.18 ± 0.15 with a minimum of seven correct answers and a maximum of 21 correct answers.

**Table II:** Answers to CPR knowledge related questions.

Questions	Responses	N	%
1. What is the abbreviation of “BLS”?	a) Best Life Support	11	3.5
	b) Basic Life Support	299	94.3
	c) Basic Lung Support	3	.9
	d) Basic Life Services	4	1.3
2. When you find someone unresponsive in the middle of the road, what will be your first response?	a) Open airway	91	28.7
	b) Start chest compression	33	10.4
	c) Look for safety	190	59.9
	d) Give two breathings	3	.9
3. If you confirm somebody is not responding to you even after shaking and shouting at him, what will be your immediate action?	a) Start CPR	153	48.3
	b) Activate EMS	82	25.9
	c) Put him in recovery position	75	23.7
	d) Observe	7	2.2
4. What is the location for chest compression?	a) Left side of the chest	29	9.1
	b) Right side of the chest	8	2.5
	c) Centre of the chest on lower half of breast bone	211	66.6
	d) Xiphisternum	69	21.8
5. What is the location for chest compression in infants?	a) One finger breadth below the nipple line	162	51.1
	b) At the intermammary line	39	12.3
	c) One finger breadth above the nipple line	31	9.8
	d) At Xiphisternum	85	26.8
6. How do you give rescue breathing in infants? a	a) Mouth-to-mouth with nose pinched	113	35.6
	b) Mouth-to-mouth and nose	98	30.9
	c) Mouth-to-nose only	19	6.0
	d) Mouth-to-mouth without nose pinched	87	27.4
7. Depth of compression in adults during CPR	a) At least 2 inches	123	38.8
	b) 2½ – 3 inches	141	44.5
	c) 1 – 1½ inches	42	13.2
	d) 1½ inch	11	3.5
8. Depth of compression in Children during CPR	a) 2 inches	66	20.8
	b) 2 - 2½ inches	45	14.2
	c) 1 - 1½ inches	107	33.8
	d) ½ – 1 inch	99	31.2
9. Depth of compression in neonates during CPR	a) 1½ – 2 inches	64	20.2
	b) 2- 2½	41	12.9
	c) 1 inch	138	43.5
	d) approximately 1½ inch	74	23.3
10. Rate of chest compression in adult and Children during CPR	a) at least 100 / min	137	43.2
	b) approximately 100 / min	81	25.6
	c) 80 / min	60	18.9
	d) 120 / min	39	12.3
11. What does abbreviation AED stands for?	a) Automated External Defibrillator	114	36.0
	b) Automated Electrical Defibrillator	144	45.4
	c) Advanced Electrical Defibrillator	40	12.6
	d) Advanced External Defibrillator	19	6.0
12. What does abbreviation EMS stands for?	a) Effective Medical Services	17	5.4
	b) Emergency Management Services	152	47.9
	c) Emergency Medical Services	130	41.0
	d) External Medical Support	18	5.7
13. If you and your friend are having food in a canteen and suddenly your friend starts expressing symptoms of choking but responsive, what will be your first response?	a) Give abdominal thrusts	121	38.2
	b) Give chest compression	26	8.2
	c) Confirm foreign body aspiration by talking to him	61	19.2
	d) Give back blows	109	34.4

**Table III:** Answers to CPR attitude related questions.

Questions	Responses	N	%
14. Do you think BLS is necessary?	Yes	310	97.8
	No	5	1.6
	Not sure	2	.6
15. If yes, how necessary it is?	Very much important	273	86.1
	Important	44	13.9
16. Have you ever voluntarily performed BLS?	Yes	147	46.4
	No	144	45.4
	Performed but not voluntarily	26	8.2
17. Would you perform mouth to mouth ventilation for person of same gender?	Yes	188	59.3
	No	71	22.4
	Hesitant	58	18.3
18. Would you perform mouth to mouth ventilation for person of opposite gender?	Yes	126	39.7
	No	97	30.6
	Hesitant	94	29.7
19. Would you like to undergo BLS training in a workshop / contre with hands on practice under supervision?	Yes	288	90.9
	No	27	8.5
	Not sure	2	.6
20. Do you think that BLS training should be a part of your curriculum?	Yes	301	95.0
	No	8	2.5
	Not sure	8	2.5

The mean number of correct answers for all CPR related questions was not statistically significantly different across the demographics. (p values > .05) (Table-V).

**Table IV:** Answers to CPR practice related questions.

Questions	Responses	N	%
21. Which of the following is not included in the 5 links in the adult Chain of Survival?	a. Early CPR	50	15.8
	b. Integrated post cardiac arrest care	99	31.2
	c. Advanced airway placement	74	23.3
	d. Rapid defibrillation	94	29.7
22. How often should rescuers switch roles when performing 2-rescuer CPR?	a. After each cycle	75	23.7
	b. After 2 cycles	157	49.5
	c. After 5 cycles	85	26.8
23. The initial Basic Life Support (BLS) steps for adults are:	a. Assess the victim, give 2 rescue breaths, defibrillate, start CPR	36	11.4
	b. Assess the victim, activate EMS & get AED, check pulse, start CPR	151	47.6
	c. Check pulse, give rescue breaths, assess the victim, defibrillate	39	12.3
	d. Assess the victim, start CPR, give 2 rescue breaths, defibrillate	91	28.7
24. Where should you attempt to perform a pulse check in adult?	a. Carotid	241	76.0
	b. Brachial	28	8.8
	c. Ulnar	44	13.9
	d. Temporal	4	1.3
25. The compression to ventilation ratio for the lone rescuer giving CPR to victims of ANY age is:	a. 15:1	44	13.9
	b. 15:2	64	20.2
	c. 30:1	41	12.9
	d. 30:2	168	53.0
26. The proper steps for operating an AED are:	a. On the AED, attach electrode pads, shock the patient, analyze the rhythm	35	11.0
	b. On the AED, attach electrode pads, analyze the rhythm, clear the patient, deliver shock	202	63.7
	c. Attach electrode pads, check pulse, shock patient, analyze rhythm	42	13.2
	d. Check pulse, attach electrode pads, analyze rhythm, shock patient.	38	12.0
27. The 2010 AHA Guidelines for CPR recommended BLS sequence of steps are:	a. Chest compressions, Airway, breathing	69	21.8
	b. Airway, Breathing, Check Pulse	91	28.7
	c. Airway, Breathing, Chest Compressions	135	42.6
	d. Chest compression, Airway placement, Breathing	22	6.9
28. Which of the following is not a sign of severity of airway obstruction?	a. Poor air exchange	83	26.2
	b. High-pitched noise while inhaling	63	19.9
	c. Unable to cry	76	24.0
	d. May wheeze between coughs	95	30.0
29. In an adult with an advanced airway in place during 2-rescuer CPR, breaths should be administered how often?	a. Every 5 seconds	76	24.0
	b. Every 5-6 seconds	113	35.6
	c. Every 6-8 seconds	70	22.1
	d. Every 10-12 seconds	58	18.3
30. The critical characteristics of high-quality CPR include which of the following?	a. Starting chest compressions within 10 seconds of recognition of cardiac arrest	47	14.8
	b. Push hard, push fast	40	12.6
	c. Minimize interruptions	38	12.0
	d. All of the above	192	60.6
31. Have you ever performed a CPR?	Yes	229	72.2
	No	88	27.8

**Table V:** Number of correct answers across the studied demographic characteristics.

Characteristics	KAP score Mean ± SD	p-value
Age of the doctors		
23-25	14.04 ± 2.78	0.211
26-30	14.65 ± 2.52	
31-34	15.57 ± 3.21	
Gender of doctors		
Male	14.38 ± 2.64	0.244
Female	14.01 ± 2.85	
Time since graduation		
Last year	14.07 ± 2.82	0.376
Last 2-3 years	14.48 ± 2.50	
Last 4-5 years	15.42 ± 2.87	
> 5 years	14.69 ± 1.54	
Doctor having valid CPR certificate		
Yes	14.36 ± 2.61	0.148
No	13.95 ± 2.91	
Time since last CPR training		
1 year or less	14.26 ± 2.67	0.090
>1 to 2 years	14.10 ± 2.86	
>2 to 3 years	11.22 ± 3.19	
>3 to 4 years	14.69 ± 3.12	
Doctors attended CPR course		
Yes	14.07 ± 2.51	.200
No	14.44 ± 3.30	

## DISCUSSION

No doctor could give 100 percent the current answer according to our study. The highest percentage of correct answer given by a doctor was 68%. This study finding goes in line with a previous Indian study which included doctors, other health workers, and medical students.^8^ Knowledge regarding infant and children CPR in comparison with the adult CPR was shown to be poorer in this study (Table-II). This might be due to the overall prevalence of children cardiac arrest cases being lower than the adult cardiac arrest cases.^9^ Only 54.6% of doctors had valid CPR certificate in 13 tertiary care hospitals in Pakistan, whereas a study revealed 99% of the medical students received CPR training in UK.^10^

Interestingly, a fewer percentage of doctors would perform mouth to mouth breathing on the opposite gender (39.7%) compared to the same gender (59.3%) (Table-III). Gender based barriers still exist in the health care provision in Pakistan.^11^ The clear majority wanted to get hands-on BLS training and suggested BLS training to be included in the medical curriculum. This study encourages medical educationists to look at this matter. Because, trained doctors show better CPR related knowledge, attitude, and practice than the untrained doctors.^12^,^13^

This study showed the duration of medical practice improves knowledge, attitude, and practice score of CPR although not at a statistically significant level (Table-V). Similar and comparable findings were shown by a Malaysian study.^14^

### Limitation of the study

The study was cross-sectional in nature therefore causality cannot be established. Data were only collected from the tertiary hospitals, which may not represent all the doctors of the country. Only the doctors were included in this study whereas CPR knowledge is essential for all the health workers. Awareness regarding ACLS protocol was not studied.

## CONCLUSION

It is unacceptable to work in a hospital as a doctor without knowing how to perform a basic life-saving procedure like CPR. The current study raises question about how to improve the knowledge, attitude and, practice among the doctors who never had CPR training. Despite showing an overall poor knowledge most of the participants wanted to perfect the CPR steps. Hospitals should provide enough resources to ensure all its health workers learn and relearn BLS protocols.

### Recommendation

This study recommends further studies to assess BLS and ACLS awareness among all health workers of the country. This study also encourages the hospitals to provide mandatory CPR training to the health workers at free of cost.

### Authors’ Contributions

**SKG, AJ & FS:** Worked on the concept and design of the study.

**MHG, FZ:** Collected the data.

**AJ:** Analyzed the data.

**SKG:** Approved the final version to be published.

## References

[1] Nag T, Ghosh A (2013). Cardiovascular disease risk factors in Asian Indian population:a systematic review. J Cardiovas Disease Res.

[2] World Health Organization (2010). World health statistics 2010.

[3] Norris RM (1998). Fatality outside hospital from acute coronary events in three British health districts 1994-5. Bmj.

[4] Rubeen R, Zareen N, Khan F, Rattan S, Moiz M, Nousheen N (2013). Awareness and Practice Of Basic Life Support Among Doctors In Civil Hospital Karachi. Medical channel.

[5] Berg RA, Hemphill R, Abella BS, Aufderheide TP, Cave DM, Hazinski MF (2010). Part 5: adult basic life support:2010 American Heart Association guidelines for cardiopulmonary resuscitation and emergency cardiovascular care. Circulation.

[6] Nambiar M, Nedungalaparambil NM, Aslesh OP (2016). Is current training in basic and advanced cardiac life support (BLS &ACLS) effective?A study of BLS &ACLS knowledge amongst healthcare professionals of North-Kerala. World J Emerg Med.

[7] Kar SS, Ramalingam A (2013). Is 30 the magic number?Issues in sample size estimation. Nat J Commun Med.

[8] Chandrasekaran S, Kumar S, Bhat SA (2010). Awareness of basic life support among medical, dental, nursing students and doctors. Ind J Anaesthesia.

[9] Meyer L, Stubbs B, Fahrenbruch C, Maeda C, Harmon K, Eisenberg M (2012). Incidence, causes, and survival trends from cardiovascular-related sudden cardiac arrest in children and young adults 0 to 35 years of age:a 30-year review. Circulation.

[10] Graham CA, Scollon D (2002). Cardiopulmonary resuscitation training for undergraduate medical students:a five?year study. Med Educ.

[11] Mumtaz Z, Salway SM (2007). Gender, pregnancy and the uptake of antenatal care services in Pakistan. Sociol Health & illness.

[12] Yunus M, Mishra A, Karim HM, Raphael V, Ahmed G, Myrthong CE (2015). Knowledge, attitude and practice of basic life support among junior doctors and students in a tertiary care medical institute. Int J Res Med Sci.

[13] Passali C, Pantazopoulos I, Dontas I, Patsaki A, Barouxis D, Troupis G (2011). Evaluation of nurses'and doctors'knowledge of basic &advanced life support resuscitation guidelines. Nurse Educ Pract.

[14] Chew KS, Hashairi FM, Zarina ZI, Farid AS, Yazid MA, Hisamudddin NA (2011). A survey on the knowledge, attitude and confidence level of adult cardiopulmonary resuscitation among junior doctors in Hospital Universiti Sains Malaysia and Hospital Raja Perempuan Zainab II, Kota Bharu, Kelantan, Malaysia. Med J Malaysia.

